# Understanding diarrhoeal diseases in response to climate variability and drought in Cape Town, South Africa: a mixed methods approach

**DOI:** 10.1186/s40249-023-01127-7

**Published:** 2023-08-18

**Authors:** Tristan Taylor Lee, Mohamed Aqiel Dalvie, Martin Röösli, Sonja Merten, Marek Kwiatkowski, Hassan Mahomed, Neville Sweijd, Guéladio Cissé

**Affiliations:** 1 Swiss Tropical and Public Health Institute, Allschwil, Switzerland; 2 University of Basel, Basel, Switzerland; 3 Centre for Environmental and Occupational Health Research, School of Public Health, University of Cape Town, Cape Town, South Africa; 4 Metro Health Services, Western Cape Government: Health and Wellness, Western Cape, South Africa; 5 Division of Health Systems and Public Health, Department of Global Health, Faculty of Medicine and Health Sciences, Stellenbosch University, Stellenbosch, South Africa; 6 Alliance for Collaboration on Climate and Earth Systems Science, Council for Scientific and Industrial Research, Pretoria, South Africa

**Keywords:** Diarrhoea, Children, Climate variability, Climate change, Water scarcity, Sub-Saharan Africa

## Abstract

**Background:**

The climate of southern Africa is expected to become hotter and drier with more frequent severe droughts and the incidence of diarrhoea to increase. From 2015 to 2018, Cape Town, South Africa, experienced a severe drought which resulted in extreme water conservation efforts. We aimed to gain a more holistic understanding of the relationship between diarrhoea in young children and climate variability in a system stressed by water scarcity.

**Methods:**

Using a mixed-methods approach, we explored diarrhoeal disease incidence in children under 5 years between 2010 to 2019 in Cape Town, primarily in the public health system through routinely collected diarrhoeal incidence and weather station data. We developed a negative binomial regression model to understand the relationship between temperature, precipitation, and relative humidity on incidence of diarrhoea with dehydration. We conducted in-depth interviews with stakeholders in the fields of health, environment, and human development on perceptions around diarrhoea and health-related interventions both prior to and over the drought, and analysed them through the framework method.

**Results:**

From diarrhoeal incidence data, the diarrhoea with dehydration incidence decreased over the decade studied, e.g. reduction of 64.7% in 2019 [95% confidence interval (*CI*): 5.5–7.2%] compared to 2010, with no increase during the severe drought period. Over the hot dry diarrhoeal season (November to May), the monthly diarrhoea with dehydration incidence increased by 7.4% (95% *CI*: 4.5–10.3%) per 1 °C increase in temperature and 2.6% (95% *CI*: 1.7–3.5%) per 1% increase in relative humidity in the unlagged model. Stakeholder interviews found that extensive and sustained diarrhoeal interventions were perceived to be responsible for the overall reduction in diarrhoeal incidence and mortality over the prior decade. During the drought, as diarrhoeal interventions were maintained, the expected increase in incidence in the public health sector did not occur.

**Conclusions:**

We found that that diarrhoeal incidence has decreased over the last decade and that incidence is strongly influenced by local temperature and humidity, particularly over the hot dry season. While climate change and extreme weather events especially stress systems supporting vulnerable populations such as young children, maintaining strong and consistent public health interventions helps to reduce negative health impacts.

**Supplementary Information:**

The online version contains supplementary material available at 10.1186/s40249-023-01127-7.

## Background

With a high disease burden in sub-Saharan Africa [1], diarrhoeal diseases are estimated to be the fifth leading cause of death in children under the age of five. Diarrhoeal diseases are known climate sensitive diseases and models predict the relative risk of diarrhoeal disease to increase by 8–11% from 2010–2039 under climate change [2–6]. The majority of the additional deaths due to diarrhoeal diseases are expected to occur in Africa[7].

Climate change disproportionately affects vulnerable groups, including children [8]. In addition to the effects of climate change on disease epidemiology (e.g. changing infectious disease incidence and distribution), it may exacerbate existing inequities, increasing poverty rates and migration, and lowering educational attainment [9]. Severe weather events are predicted to become more frequent and intense [10]. Droughts have been identified as the climate event that is not only very costly, but has the greatest consequence on the population and results in the highest mortality [11].

Diarrhoeal diseases are acquired through multiple exposure pathways that are linked to Water, Sanitation, and Hygiene (WaSH) practices [12] and the aetiology is highly site specific [13, 14]. In the Western Cape province in 2016, the self-reported prevalence of diarrhoeal disease in the previous 2-weeks in children under five was 5% [15]. A household survey conducted in formal and informal settlements in Cape Town between March and June 2017 found that the 1-week prevalence of diarrhoeal disease in children under five was 15.3% [16]. Given that in the Western Cape, diarrhoeal disease occurs seasonally over the hot and dry period (November–May) [17] and therefore, it is likely that incidence is driven by seasonal climate characteristics or related behaviours. In Cape Town, stool samples from children under twelve presenting to clinics with diarrhoea had diarrhoeic *Escherichia coli*, *Shigella flexneri*, *Plesiomonas shigelloides*, *Salmonella enterica*, *Campylobacter jejuni*, and *Aeromonas sobria*, and these diarrhoea-associated pathogens were also present in the environment (eg. surface water from the local river and raw meat from road-side stalls) [18]. Rotavirus vaccine coverage was 67% (two doses) in the Western Cape in 2016 [15].

Research has shown that increased temperature is positively associated with increased incidence of bacterially caused diarrhoeal disease by systematic review [4, 5]. In Limpopo province of South Africa, hospital admissions for diarrhoeal disease in children under five increased with drier and hotter than average periods in the dry winter season, as well as with higher than average precipitation during the rainy season [19]. In Cape Town, Musengimana et al. (2016) evaluated the effect of temperature on diarrhoeal disease incidence for the peak diarrhoeal disease season in 2012 and 2013 and determined that a 5 °C increase in minimum and maximum temperature results in a 32–40% increase in diarrhoeal disease incidence for the two years studied [17]. Precipitation likely impacts disease incidence through the concentration-dilution mechanism, where low rainfall allows pathogens to concnetrate in the environment and high rainfall dilutes or flushes them. Bacterial and parasitic diarrhoea appears to be more frequent during rainy seasons [3]. Little research on the relationship between drought and diarrhoeal disease has been conducted to date, although multiple researchers suspect a positive association [3, 5, 20]. One case–control study during drought on the island country of Tuvalu found that having a low water tank level and reduced hand washing led to increased odds of having diarrhoeal disease [21]. Overall, it is likely that weather and climate influences diarrhoeal disease by creating conditions which enhance or impede pathogen proliferation and survival as well as effecting human behaviours and environmental exposure [3–5, 22, 23].

In Cape Town, a severe drought occurred over the hydrological years of 2015–2017 (November 1, 2015–October 31, 2018). Consecutive years of low rainfall, in combination with other climatic and human factors, resulted in the rainfed water supply not being replenished over the winter season [24]. In response to the drought, the government implemented conservation efforts including severe water restrictions, punitive tariffs, water consumption management devices, water pressure reduction, behavioural nudges (e.g. letters, a map publicly displaying water usage by household), irrigation reduction, education campaigns, and the threat of sealing non-essential water taps [25–27]. As a result of this imminent threat, public water supply use dropped by half over the 2.5 year drought [26].

The major concern with diarrhoeal disease due to the expected increase in frequency of days > 35 °C in the Western Cape, decrease in annual rainfall, increased frequency and severity of drought resulting from climate change impacts [11, 28, 29], and potential changes in WaSH practices warrants further research. This study uses a multipronged approach to understand trends in severe diarrhoeal disease cases (diarrhoeal disease with dehydration) in children under the age of five in Cape Town, in response to climate variability, as well as in times of water scarcity during the hot dry season where diarrhoeal disease incidence is generally higher. We performed a negative binomial regression to understand the effect of climate variability, especially in light of the Cape Town drought, on the incidence of diarrhoeal disease with dehydration in Cape Town. Additionally, as diarrhoeal disease rates may also be influenced by health system interventions, we conducted in-depth interviews with stakeholders about diarrhoeal disease, and health interventions before and during the drought.

## Methods

### Study setting

In 2019, the population of Cape Town was 4.2 million people, of which 336,000 were children under the age of five [30]. In terms of social determinants of health, in 2016, 81.6% of the population resided in formal dwellings and the average household size was 3.2 people. At the household level, 91.0% had a flush toilet connected to sewerage, 87.8% had weekly refuse removal, 76.7% had piped water inside of the dwelling, and 97.2% had electricity for lighting [31]. The unemployment rate in 2018 was 22.4% [32].

**Fig. 1:**
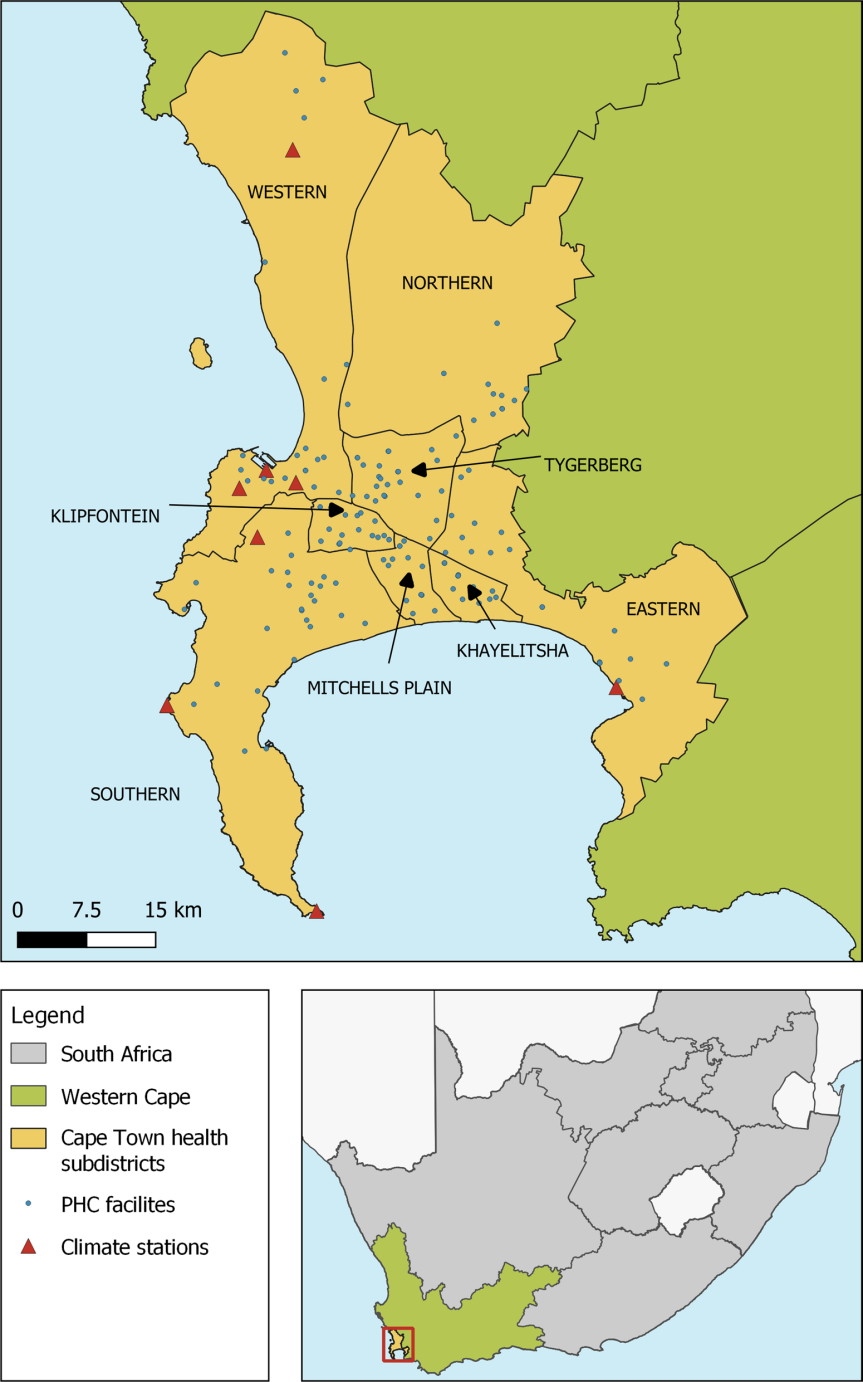
Map of Cape Town showing health subdistrict boundaries, locations of weather stations, and primary health care facilities. City health region data was downloaded as a shape file from the City of Cape Town Open Data Portal. Primary health care facilities coordinates were given by the Western Cape department of health and weather station coordinates were given in the weather data from the SAWS. The figure was generated in QGIS and projected on the WGS 84/UTM zone 34S (EPSG:32734) coordinate reference system. SAWS: South African Weather Service, PHC: Public Healthcare

In Cape Town, the public health system consists of the provincial Department of Health, which primarily provides curative treatment with clinics and hospitals, and City Health (municipal), which provides primary care and some curative services, including environmental health services, in addition to running some clinics. The provincial health data divides the city into eight subdistricts; Northern, Eastern, Khayelitsha, Mitchells Plain, Southern, Klipfontein, Tygerberg, and Western (Fig. 1). In the Western Cape, the public health system serves the majority of the population; in 2018, only 25.1% of individuals had medical insurance [33].

### Study design

A mixed methods approach was used to characterize the situation around diarrhoeal disease in children under five in Cape Town. We used routinely collected weather data and diarrhoeal disease data from the public health system (i.e. Western Cape department of health). We also conducted in-depth interviews on perceptions on diarrhoeal disease and drought in Cape Town. In the subsections below, we provide the details of the data collection specifications and statistical methods employed.

### Data collection

The health dataset consists of the monthly case count of diarrhoeal disease with dehydration, both moderate and severe cases, by facility, in children under five who were diagnosed at primary health care facilities in the Western Cape from 2010 to 2019. This data is quality controlled and compiled in an anonymized aggregate format by the provincial and local (City Health) department of health in the Standard Information Jointly Assembled by Networked Infrastructure (SINJANI) database and was accessed through the National Health Research Database platform for South Africa. Children who did not report to a facility or those that reported to a private facility are not captured in this dataset. The data was filtered for primary care facilities. Cases recorded at hospitals were excluded to avoid double-counting. The regression analysis was restricted to the health district of the Cape Town due to differences in lifestyles (e.g. urban vs rural, care-seeking behaviours). Data on socioeconomic factors are not recorded in this dataset. Annual population estimates for children under five in the Western Cape by subdistrict was collected from the Western Cape Government department of health.

Weather data on daily maximum and minimum temperature, precipitation, and relative humidity at 8:00, 14:00, and 20:00 o’clock was collected from the South African Weather Service (SAWS) (www.weathersa.co.za) for 2010–2019 from weather stations in the Cape Town area (Fig. 1).

Key stakeholder interviews were conducted with personnel working in the Western Cape in the fields of health, environment, and human settlements to understand perceptions around diarrhoeal diseases and related health interventions in Cape Town. A semi-structured interview guide with open questions was used to gather ideas and further probe for information. The target sample size of 13–17 interviews was selected to reach theoretical saturation, where no new themes emerge and no new insights are gained with subsequent interviews. Purposive and snowball sampling was used, with potential interviewees identified through the South African collaborators and interviewees themselves. Additionally, a research application was submitted to City Health to speak with government officials. The resulting approval specified four participants available for contact.

Participants were recruited by email invitation. Written informed consent was obtained from all interviewees. No compensation was offered. A pilot interview was conducted as part of training and to refine the interview guide questions and prompts. The interview guide was discussed with TL, SM, and GC prior to conducting interviews. All interviews were conducted in English in Cape Town between November and December 2019 by TL. With permission, each interview was audio recorded and hand-written notes were taken. Interviews were transcribed by TL and checked for accuracy through listening to the interviews while reviewing the transcripts. Participants were aware of their ability to withdraw from the study at any time.

### Statistical analysis

The prevailing climate (temperature, precipitation, and humidity) of each subdistrict was derived from weather station data by taking the mean value of each weather parameter of the weather stations within the subdistrict, or, if the subdistrict lacked a weather station, the nearest weather station to the geometrical centre of the subdistrict. This was done using the ‘Nearest Neighbour’ function in QGIS software (v3.4, QGIS.org, 2018. QGIS Geographic Information System. QGIS Association. http://www.qgis.org). Missing data for all variables accounted for approximately 2.2% of the database and were subsequently filled in by linear interpolation. Data from the Royal Yacht Club weather station was excluded due to 6.5 consecutive months of daily missing data (April–October 2011). However, this station is situated in the same subdistrict (Western) as both the Observatory and Molento Reservoir stations, and its readings could be predicted well using linear regression on these stations (data not shown). Monthly averages of daily maximum and minimum temperature, daily precipitation, and humidity at 8:00, 14:00, and 20:00 o’clock were calculate for each station. In general, the humidity at 14:00 o’clock approximates minimum humidity while the 8:00 and 20:00 o’clock humidity values are similar and approximate the maximum humidity.

A negative binomial regression model with the log link function was developed using “population under five” as an offset, weather parameters as explanatory variables, and diarrhoeal disease with dehydration cases per month per subdistrict as the outcome. The data was analyzed at the subdistrict level and by month because the population data was available at the subdistrict level and the diarrhoeal disease with dehydration incidence data was only available as monthly aggregates. Subdistricts were used as fixed predictors. The lagged Pearson’s residual was also included to account for serial autocorrelation of errors. In Cape Town, the diarrhoeal surge season, which occurs during the summer season between November and May, is the primary period of interest due to the high diarrhoeal disease burden on the health care system. For this reason, only the diarrhoeal disease surge season was included in the model. Restriction of the model to the surge season also reduces the range in predictor values such that a linear model can be used as an approximation for the data. Of the weather covariates listed below, only a few were selected to minimize collinearity. These were maximum temperature, mean humidity at 8:00, and average precipitation. The effect of climate variability on diarrhoeal diseases with dehydration is reported as incidence rate ratios (*IRR*) with 95% confidence intervals (*CI*). As a descriptive analysis of monthly aggregated diarrhoeal disease incidence did not show any major increases or decreases, as well as the height of the drought and water restrictions occurring after the usual peak in diarrhoeal disease incidence, we did not feel that other analysis methods were warranted. To account for potential effects of the drought, year was included as a covariate in regression models. Data was analyzed using R 4.0.2 (Lucent Technologies, Jasmine Mountain, USA).

Interviews were analyzed using the framework method [34], whereby themes were developed from research questions as well as participants’ responses. Transcribed interviews were coded and analyzed in MaxQDA 2018 (VERBI Software, Berlin, Germany). Codes and themes were discussed between the researchers TTL, SM, and GC. A codebook with definitions of each code was also used to ensure consistency while coding interviews (Additional file 2: Table S1). After the iterative coding process, a code tree (Additional file 1: Fig. S1) was constructed based on the codes, categories, and themes observed in the interviews. Interview data was populated into this framework and used for analysis (Additional file 2: Table S2).

## Results

### Description of diarrhoeal disease with dehydration and climate variability trends

**Fig. 2:**
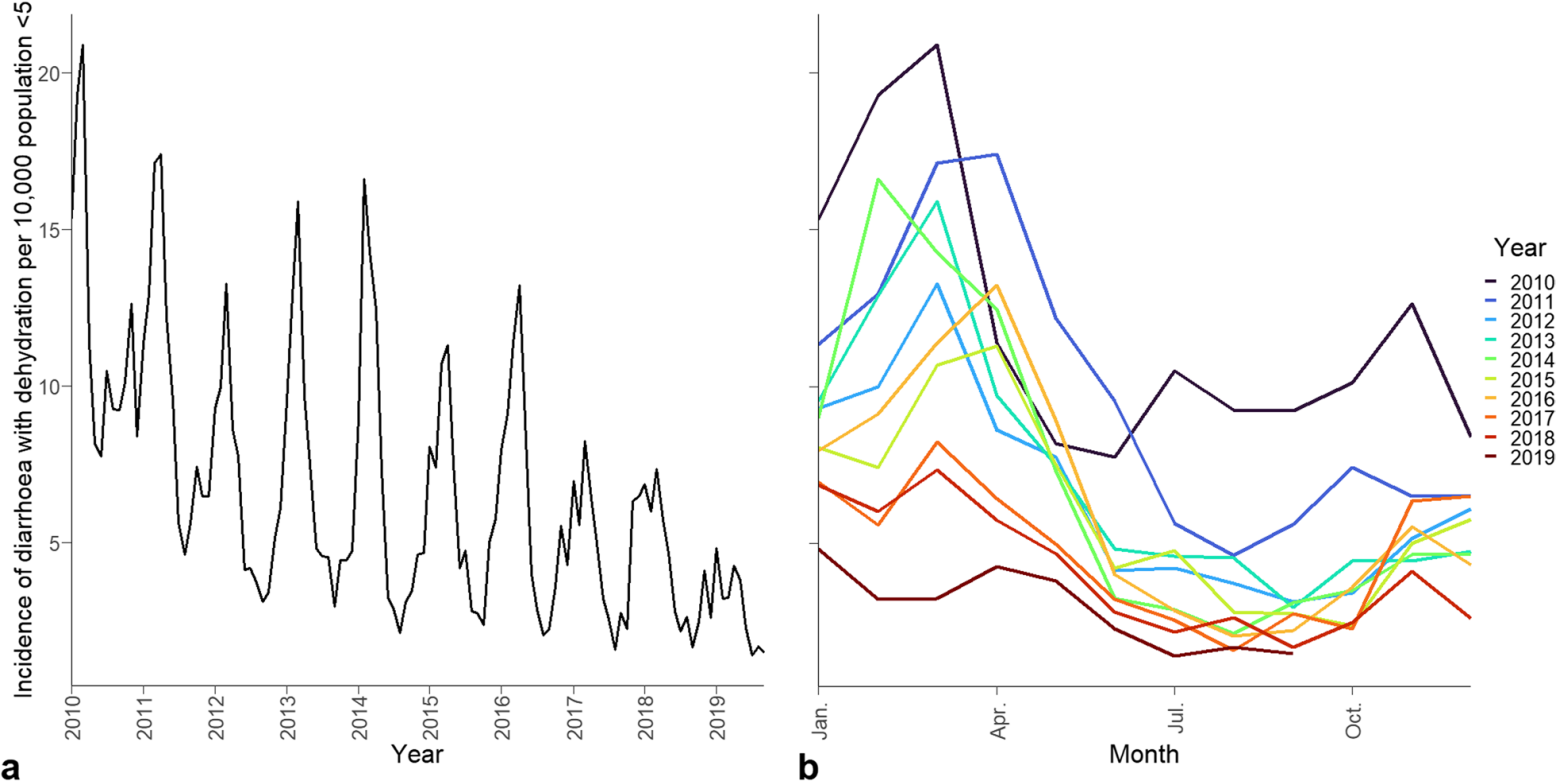
Monthly population adjusted distribution of diarrhoeal disease with dehydration in Cape Town in children under five shown **a** across the study period and **b** by year

Within the Western Cape Province, 80.3% of all diarrhoeal disease with dehydration cases recorded in primary health care facilities from January 2010–September 2019 occurred in the Cape Town district, where diarrhoeal disease with dehydration rates triple during the diarrhoeal surge season (Fig. 2). The peak month for diarrhoeal disease with dehydration occurs between February to April, depending on the year. This trend is present in all years, even though the peak diarrhoeal burden has been steadily decreasing over the period analysed. From 2010–2019, 19,795 of diarrhoeal disease with dehydration cases occurred during the diarrhoeal surge where the minimum, median, and maximum cases of diarrhoeal disease with dehydration recorded in a subdistrict in a month were 0, 21, and 236, respectively. When disaggregated to the subdistrict level, the diarrhoeal disease with dehydration rates decrease in all subdistricts over the 10 years assessed, with the overall cases being highest in Northern and Khayelitsha, with incidence rates of 10.2 and 9.9 cases per 10,000 children under five, respectively.

**Fig. 3:**
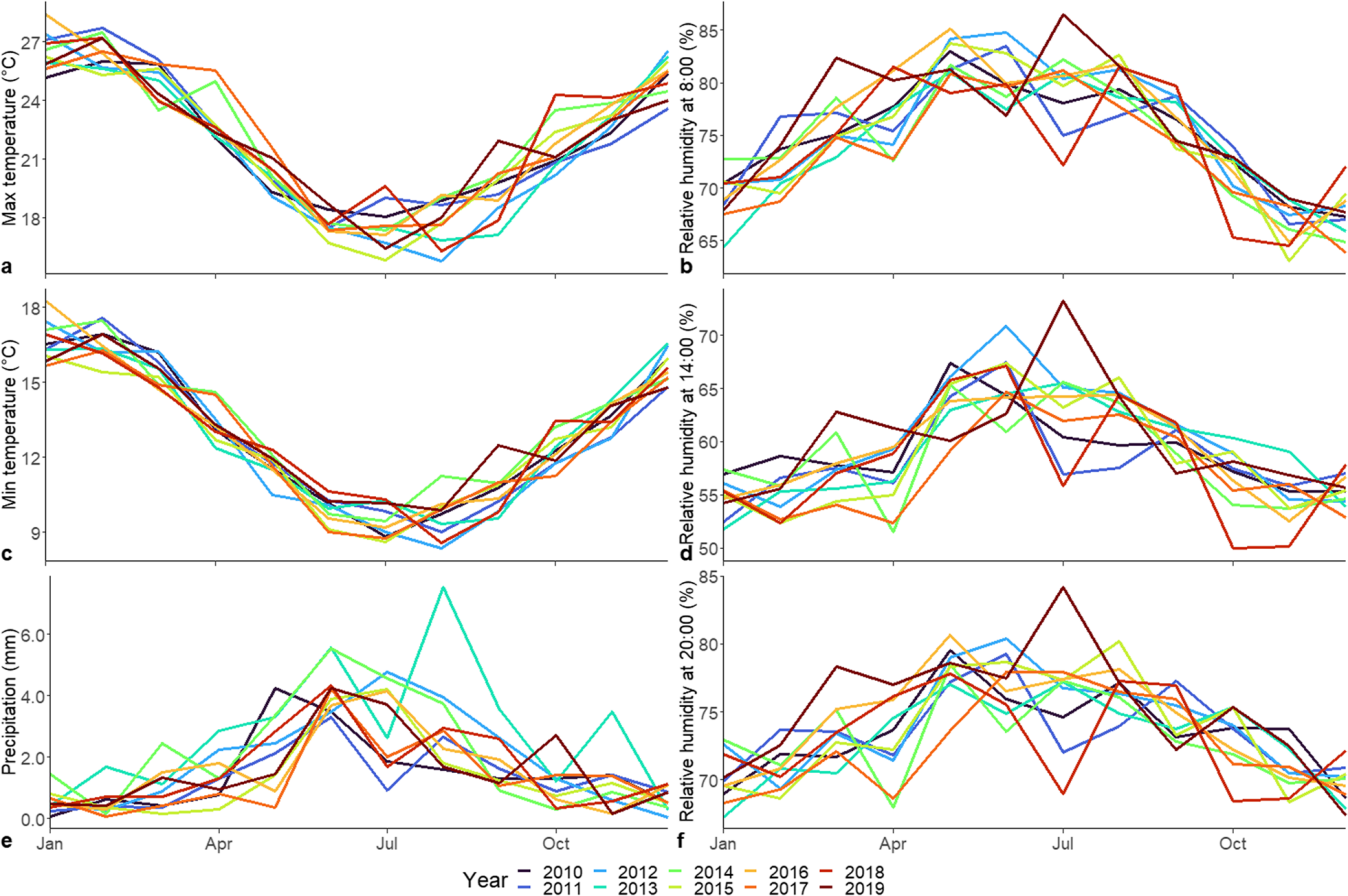
Distribution of mean monthly weather parameters across the year in Cape Town between January 2010–Dec. 2019. Monthly values are derived by averaging daily measurements from seven SAWS weather stations in the Cape Town for **a** mean maximum temperature, **b** mean minimum temperature, **c** mean precipitation, **d** mean relative humidity at 8:00, **e** mean relative humidity at 14:00, and **f** mean relative humidity at 20:00

The location of SAWS weather stations in relation to subdistricts and weather variability over time are shown in Fig. 1 and Fig. 3. Of note, the stations Cape Point and Slangkop are located close to large water bodies and this is reflected in the low maximum temperatures and high and constant relative humidity throughout the day (Additional file 1: Figs. S2, S3). In the precipitation data, the Kirstenbosch station recorded a much higher average precipitation than the remaining stations due to orographic precipitation at this location (Additional file 1: Figs. S2, S3). In relation to weather, the diarrhoeal disease with dehydration rate decreases irrespective of temperature and humidity trends and the peak in incidence visually appears to be lagged by approximately one month to the peak in maximum temperature (Additional file 1: Fig. S4).

### Regression analysis of climate variability on diarrhoeal disease with dehydration incidence

The adjusted negative binomial regression analysis with weather variables of the same month (no lag) shows that maximum temperature and relative humidity at 8:00 are significantly positively associated with incidence of diarrhoeal disease with dehydration, with *IRR*s of 1.074 (95% *CI*: 1.045–1.103) and 1.026 (95% *CI*: 1.017–1.035), respectively (Table 1). This signifies an increase in diarrhoeal disease with dehydration of 7% for each 1 ˚C increase in temperature and 3% increase in diarrhoeal disease with dehydration for each 1% increase in relative humidity during the diarrhoeal surge season. Mean precipitation was not significantly associated with incidence of diarrhoeal disease with dehydration [*IRR* = 0.963 (95% *CI*: 0.893–1.038)] during the diarrhoeal surge season. The subdistricts Khayelitsha [*IRR* = 1.893 (95% *CI*: 1.579–2.269)] and Northern [*IRR* = 1.745 (95% *CI*: 1.437–2.118)], in particular, showed higher rates of diarrhoeal disease with dehydration compared to the other subdistricts.

**Table 1:** Population adjusted negative binomial regression model for diarrhoeal disease with dehydration cases in primary health care facilities in Cape Town, South Africa

Parameter	No lag		Lag 1-month	
	Incidence rate ratio (95%* CI*)	*P*-value	Incidence rate ratio (95% *CI*)	*P*-value
Mean max temperature	1.074 (1.045–1.103)	< 0.001	1.107 (1.080–1.135)	< 0.001
Mean precipitation	0.963 (0.893–1.038)	0.31	1.029 (0.949–1.117)	0.47
Mean 8:00 relative humidity	1.026 (1.017–1.035)	< 0.001	1.016 (1.007–1.025)	< 0.001
*Subdistrict*				
Eastern	Ref	Ref	Ref	Ref
Khayelitsha	1.893 (1.579–2.269)	< 0.001	1.854 (1.554–2.211)	< 0.001
Klipfontein	1.328 (1.097–1.608)	0.003	1.382 (1.147–1.665)	< 0.001
Mitchell’s Plain	1.049 (0.868–1.268)	0.61	1.140 (0.948–1.372)	0.16
Northern	1.745 (1.437–2.118)	< 0.001	1.824 (1.515–2.197)	< 0.001
Southern	1.290 (1.055–1.577)	0.012	1.570 (1.269–1.943)	< 0.001
Tygerberg	1.103 (0.913–1.333)	0.30	1.164 (0.968–1.400)	0.10
Western	1.159 (0.961–1.398)	0.12	1.224 (1.020–1.469)	0.027
*Annual trend*				
2010	Ref	Ref	Ref	Ref
2011	1.093 (0.879–1.359)	0.41	0.938 (0.747–1.177)	0.57
2012	0.807 (0.649–1.004)	0.049	0.775 (0.618–0.972)	0.024
2013	0.896 (0.718–1.119)	0.32	0.750 (0.593–0.949)	0.014
2014	0.887 (0.713–1.104)	0.27	0.732(0.581–0.923)	0.007
2015	0.720 (0.577–0.897)	0.0028	0.678 (0.540–0.851)	< 0.001
2016	0.731 (0.586–0.913)	0.0047	0.658 (0.524–0.827)	< 0.001
2017	0.624 (0.500–0.780)	< 0.001	0.557 (0.443–0.701)	< 0.001
2018	0.462 (0.370–0.577)	< 0.001	0.412 (0.327–0.519)	< 0.001
2019	0.353 (0.275–0.452)	< 0.001	0.318 (0.247–0.410)	< 0.001

We built also an analogous model, where the weather variables were replaced with their lagged values (1 month lag). This model shows similar climate variability trends in that the effect of maximum temperature and relative humidity on diarrhoeal disease with dehydration incidence are significant and positively associated with *IRR*s of 1.107 (95% *CI*: 1.080–1.135) and 1.016 (95% *CI*: 1.007–1.025), respectively, while average precipitation is positive, but not significant [IRR: 1.029 (95% *CI*: 0.949–1.117)]. The general trend and order of magnitude of the IRRs for subdistricts were roughly similar. Similarly, the IRR decreased as the years progressed. Although lagged effects of weather variables on diarrhoeal disease with dehydration are very plausible, they likely occur on timescales of less than 1 month, and are best studied with daily data.

### Description of interview participants

As shown in Table 2, the in-depth interview sample includes fourteen participants working in the Western Cape over the drought and is primarily comprised of local and provincial government officials (*n* = 12), as well as several physicians (*n* = 3) and researchers (*n* = 4). Several stakeholders held multiple positions (e.g. physician at a public hospital and university lecturer). In terms of field of professional expertise, stakeholders’ primary focus can be heuristically grouped in several categories: general health (*n* = 10), specifically child health (*n* = 5), human development (*n* = 5), and the environment (*n* = 7). Many stakeholders worked across disciplines (e.g. Environmental health practitioners working to improve sanitation through environmental monitoring to reduce health impacts).

**Table 2:** Characteristics of main areas of work and role of interview participants

Pseudonym	Field of work^a^				Position^b^		
	Health	Child health or diarrhoea	Human development	Environment	Government official	Physician	Researcher
B	X		X	X	X		
C	X	X			X	X	X
D	X					X	X
E	X			X	X		
F			X		X		X
G			X		X		
H	X		X	X	X		
I	X			X	X		
J	X	X		X			X
K	X				X		
L	X	X			X	X	
M	X		X	X	X		
N	X	X		X	X		
O	X	X			X		

^a^X indicates that the interviewee works in the indicated field

^b^X indicates that the interviewee is employed as/affiliated with the indicated field

### Perceptions on diarrhoeal disease trends in Cape Town

Over the last decade, stakeholders have noticed a gradual decrease in diarrhoeal disease. This started with a sharp drop in mortality before a more recent decrease in incidence. One health programme manager commented that a larger proportion of sick children now present with diarrhoea without dehydration compared to diarrhoeal disease with dehydration.

Stakeholders ascribe decreases in mortality and morbidity to activities implemented during the diarrhoeal surge season (described later), addition of the national immunization programme for rotavirus, greater awareness of WaSH practices, and diarrhoeal disease in the community, better standards of living, and wider delivery of health services (including training of healthcare workers, availability of diarrhoea case management services). Although stakeholders were generally concerned about the effect of climate change on waterborne diseases, none explicitly discussed the role of climate in bringing down the incidence in diarrhoeal disease.

The strong seasonality of diarrhoeal disease in Cape Town was known to all stakeholders. Stakeholders could describe the months of the surge season response (November to May) and the peak in February/March. Many governmental stakeholders, especially those working around health, were aware of the existence or had access to the disseminated diarrhoeal disease trend summaries collected through the public health database. One paediatrician and diarrhoeal disease specialist in SA additionally described a gradual shift in peak diarrhoeal disease incidence during the surge season, from late December in the 1960s and 1970s to the peak in February/March seen today (C). Another paediatrician noted that children with recurrent bouts of diarrhoeal disease start presenting with malnutrition later in the diarrhoeal surge season (L).

When asked to describe reasons for the observed seasonal diarrhoeal disease trends in Cape Town, stakeholders often attributed peaks to the hot and dry summer weather (and more favourable pathogen proliferation conditions) and its relation to human behaviour [e.g. availability of work, food safety (e.g. lack of refrigeration in informal settlements), recreational activities]. One environmental researcher working in water quality in and out of informal settlements attributed the peak in late summer being due to concentrated pollutants being mobilized by the later summer/autumn rain, "[mess is] just lying there in in the dry summer heat. Until the first little bit of rain—this is the concentrating [part]—and [the rain] mobilizes it. It makes it move down the sidewalk, into the rivers, everywhere. It brings it into solution so to speak" (J).

In terms of short-term trends, two stakeholders working directly as paediatricians or with child health programmes noted a correlation between extremely hot days and an increase in diarrhoeal disease. However, the pattern of hot days and lag for incidence of diarrhoeal disease appeared to differ. While one stated "it'll be like 35 to 40 °C like 3 days in a row we know that we're going to have lots of diarrhoea the next day" (L), the other stated “if it has been extremely hot like from 28 °C to over 30, 32, 34 °C, 2–3 days later we see a high number of children presenting diarrhea [with and without dehydration] into our facilities" (O).

### Perceptions on broader health issues and diarrhoeal disease trends during the Cape Town drought

#### Health concerns during drought

During the drought, many were concerned for diarrhoeal disease in terms of increasing severity and incidence. This resulted in a heighted vigilance around the disease [e.g. nervousness that the drought would make things worse (I)]. One experienced Environmental Health Practitioner with a long history of involvement in diarrhoeal disease programmes primarily sees diarrhoeal disease as a problem only if risk factors are allowed to persist (e.g., M: "if we allow the accumulation of waste, and poor sanitation practices, if we allow poorer hygiene practices in restaurants… there is a risk"). There were also many broader concerns about related health issues as a consequence of water scarcity, such as increasing incidence of diseases with similar transmission routes like typhoid and hepatitis A, dehydration, malnutrition (e.g., C: "an episode of diarrhoea in a small child has a large potential to be the beginning of a slippery nutritional slope in into malnutrition, which then has a whole lot of other health consequences"). Other stakeholders were more concerned about drought’s effect on ability to provide health services (e.g., having water for rehydration of dehydrated children and the ability to keep hospitals clean) (e.g. J: "[s]o water scarcity impacts cleanliness across the board, if I can put it like that, it also impacts on the hospital's ability to keep itself clean. So transmission there becomes a higher risk as well").

#### Diarrhoea and other health trends during drought

Regarding documented trends drought, perceptions of diarrhoeal disease trends during the drought were heavily influenced by knowledge of the public health system diarrhoeal disease data. Many governmental officials were aware of the SINJANI system diarrhoeal disease statistics in children under five showing no increase in cases over the drought (or could not remember major incidences where diarrhoeal disease became a problem). One states that the drought did not seem to have a significant impact on health in the communities; no outbreaks of typhoid, diarrhoeal disease, or other waterborne diseases were noted (N). This trend may not be unique to the Cape Town drought. One stakeholder who had transitioned to working in a health zone north of Cape Town with an ongoing drought at the time of the interview noted that there was no increase in cases at the referral hospital in Paarl compared to previous non-drought years (I). Others who were not privy to this department of health dataset assumed that diarrhoeal disease may have increased based on ways people were restricting their water use, using alternative water sources with poorer water quality, and high temperatures. Several participants, both within and outside of the government/health sector felt that patterns may have been different in mid/high income groups who drastically changed their water consumption practices and did not report to public primary health care facilities.

#### Explanations for perceived diarrhoeal disease trends

Explanations for a lack of increase in diarrhoeal disease include many years of sustained diarrhoeal disease interventions, the fact that Day Zero did not happen and potable water access never became an extremely critical issue (e.g. taps did not get turned off), misalignment of the peak in diarrhoeal disease during the diarrhoeal surge season and the height of water restrictions, and hardly any changes in water use practices in vulnerable communities where the dataset is primarily captured. Some participants feel that diarrhoeal disease would have been a major issue if the sanitation systems had failed [e.g. without sanitation, more flies will breed; lack of sanitation also creates the potential for chaos and impacts food production and food safety (M)]. At the household level, concerns include poorer WaSH practices due to using less water, generally high temperatures during the drought, restricted access to water of good quality, and questions about the equivalency of alternative practices (e.g., alternative water sources, using alcohol based sanitizer). Several believe changes in diarrhoeal disease incidence and severity would be higher in high vs. low-income earners since high income groups altered their water usage much more than people living in informal settlements.

#### Interventions around diarrhoeal disease in Cape Town

Interventions around diarrhoeal disease are multi-pronged and have existed in the Western Cape for many years. In communities, education campaigns are conducted by community health workers about diarrhoeal disease and other diseases so the public learns the severity of diarrhoeal disease, signs of disease, when to seek care, as well as potential problems with using traditional medicine. Environmental Health Practioners also play a major role in preventative health by responding to specific needs, especially in vulnerable settings such as informal settlements (e.g., B: they act as the "eyes and ears on the ground of whatever issues are there"). Cases resulting in death, or moderate or severe diarrhoeal disease are investigated by a community health worker or EHP, respectively, through a house visit to observe potential health risks (e.g. N: "to go and follow up, see how is the home, uh, cleaned how are they cooking, do they have electricity, do they have access to water, and this community worker would have a one-on-one session advising that mother [on good WaSH practices]”) as well as reporting on environmental risks in the community including “the blocked drain and overflowing drain water that is just running, um, dead dog that's lying on the road” (N). Furthermore, as part of the diarrhoeal surge season response, surge meetings are conducted between multiple stakeholders within and outside of health departments to plan for the upcoming diarrhoeal surge season and review data. Plans also include setting up rehydration corners in clinics and monitoring and tracking incidences at a higher frequency (i.e. weekly) compared to outside of the diarrhoeal surge season (i.e. monthly). Local meetings may also be conducted frequently in specific communities between representatives including clinics, Environmental Health Practioners, and hospitals.

In general, interventions on diarrhoeal disease in Cape Town are viewed as highly effective; many stakeholders attributed sharp decreases in mortality and morbidity to the surge season interventions. Additionally, one stakeholder felt that general diarrhoeal disease and child health promotion activities were successful due to their targeted nature towards communities (e.g. O: I would say those interventions worked, because it's a one on one, and this is a local woman or man that is coming, that is giving health advice, in an understandable manner, in an understandable language"). However, several participants comment that the diarrhoeal disease incidence data collected is “suboptimal” (e.g. poor surveillance, lack of pathogen identification, gaps in data from hospitals and private facilities, inaccurate data provided by patients).

#### Interventions targeted towards diarrhoeal disease during drought

With regards to actual interventions that were implemented against diarrhoeal disease as a response to the drought, little seems to have changed from non-drought years. Normal campaigns continued since they were found previously to be successful. Additionally, care was taken to not reduce messages around hygiene and may have been more strongly pushed during the drought (e.g. N: "if anything at all, the messaging became stronger, um, on hygiene practices"). To pregnant women, more education was given on risks of diarrhoeal disease as well as promoting breastfeeding (i.e. where water quality is not a concern). Other interventions include training more staff on using rehydration equipment to prepare for potential upswings in cases, strengthening community health workers, improving disease surveillance to be more responsive, and monitoring diarrhoeal disease in adults near the end of the drought. One participant outside of government did not see any changes around diarrhoeal disease interventions during the drought and comments that the recommended water usage volumes were too little to maintain proper hygiene “so they just left it” (J).

## Discussion

This study uses a mixed-method approach to characterize diarrhoeal disease incidence over a ten-year period during the hot dry diarrhoeal season, which covers periods of water scarcity, in relation to climate variability. To further explore the issue of water scarcity, health interventions in place prior to and during the drought are also investigated. We found that in the context of a decreasing diarrhoeal disease with dehydration burden during the diarrhoeal surge season in Cape Town, diarrhoeal disease with dehydration was positively associated with maximum temperature and high relative humidity both with and without lag. During the drought years, no cases in addition to the seasonal surge in diarrhoeal disease were detected in this data. Sustained diarrhoeal disease interventions are seen as effective in lowering the diarrhoeal disease burden in Cape Town.

Our results contribute to the increasing body of work on diarrhoeal disease in the context of climate variability. In terms of temperature, which has a well-established relationship with diarrhoeal disease incidence, our findings support the positive association between increasing temperature and increasing incidence [4, 5]. Importantly, the results from our main model, which predicts a 37% increase in diarrhoeal disease with dehydration incidence for a 5 °C increase in mean daily maximum temperature, are in line with prior research in Cape Town [17]. The fact that temperature has an effect on diarrhoeal disease was also reflected by our interview participants, with many commenting about the hot dryness of summer contributing to diarrhoeal disease. Only one talked about the relationship with first rainfall mobilizing accumulated pathogens in late summer. While we investigated climate effects at a more aggregated level, like many other studies which have used lag times on the weekly to 1-month scale [4, 19, 35, 36], it is likely that effects at shorter lags, for example, on a daily scale [37, 38], may play significant role. This observation was captured by two interview participants working more closely with communities and patients, who noted that a sudden increase in temperature results in a sharp increase of diarrhoeal disease several days later.

Our study did not find evidence for an association of diarrhoeal disease with dehydration with rainfall. In the literature, the effect of precipitation on incidence of diarrhoeal disease varies and is measured differently. Some argue that through the concentration-dilution hypothesis, pathogens may being released upon rainfall and thus increase the incidence of diarrhoea, while other arguments suggest that rainfall may improve access to WaSH practices and thus lead to a decrease in incidence [3, 39]. Several studies have discussed the importance of interpreting precipitation in relation to season as well as to pathogen seasonality [4, 13]. However, routine diagnosis of the pathogenic agent is not conducted in Cape Town and this analysis focused on the summer season where diarrheal cases are highest and there is relatively less rainfall.

Regarding relative humidity, our study found a positive association of 8:00 am relative humidity and diarrhoeal disease incidence in both the unlagged and 1-month lagged model. One study in Taiwan, China which also found a positive association attributed this to the interlinked effects of high relative humidity and extreme rainfall events [40]. Other studies in Ethiopia, Japan, and Bangladesh have found negative associations and the finding may be explained by some diarrhoeal pathogens, including rotavirus, persisting for more than ten days at low humidity [41–43].

Overall, evidence from both the descriptive statistics and interview participants show that the incidence of diarrhoeal disease with dehydration in Cape Town has decreased over the ten years studied. In the Western Cape, mortality from diarrhoeal disease in children under five was also found to have decreased by 71% over approximately the same period (2009–2016) [44]. In Cape Town specifically, only six diarrhoeal disease deaths (corresponding to a case fatality rate of 0.3%) were reported in 2017/2018 fiscal year [30]. Interview participants, especially those working in child health for many years, noted an initial drastic decrease in mortality followed by a more recent decrease in morbidity which they attributed to the success of diarrhoeal disease interventions over the peak surge season. The shift in peak diarrhoeal disease month in the Western Cape noted by participants was also found in one hospital in Cape Town tracking diarrhoeal disease admission rates from 1976 to 2015, where the peak shifted from January to March [45].

In spite of sparsity, the current evidence and perception of drought effects in the literature points to positive relationship between rates of diarrhoeal disease and drought [3, 21]. However, this hypothesis was not confirmed by this study, as the annual diarrhoeal disease incidence in Cape Town did not increase during drought. Although the diarrhoeal disease with dehydration peak appeared to be slightly broader in 2017 and 2018, the overall number of cases is low. Due to complex definitions of drought [46] often relying on anthropogenic ideas (e.g. dam levels) and climate trends (e.g. temperature or precipitation), drought could not be accurately reflected as an additional variable in the regression model. Interview participants also did not identify any outbreaks of waterborne diseases during the drought. This was attributed to household water never being shut off during the drought, critical WaSH practices not being impacted, and timing of the drought and water restrictions being after the highest peak in diarrhoeal disease incidence. To more thoroughly assess the effect of drought on diarrhoeal disease incidence, research needs to include data on multiple droughts that occur not only hotter dryer seasons but also colder seasons. It is also important to note that this study is based on public health sector data. No data could be collected from the private sector, although some interviewees reported anecdotal evidence of increased risky WaSH behaviours and diarrhoeal disease increasing. It should be noted that in the private sector, among children with higher living standards may have better treatment seeking but the severity of cases may not have progressed to diarrhoeal disease with dehydration. It is also critical to note that during the drought, few diarrhoeal disease measures were put in place other than emphasizing hygiene measures. The sustained nature of routine diarrhoeal surge season activities ongoing during the drought may have strongly prevented outbreaks form occurring.

Our research examines severe diarrhoeal disease over a decade in a temperate climate where disease incidence peaks in the hot dry season. We account for local weather to produce incidence rate ratio estimates for three critical weather parameters which may help to inform local decision making as well as contribute to the evidence on the effect of climate on diarrhoeal diseases. The qualitative in-depth interviews of the mixed methods study design help to explain and support some of the findings of our logistic regression, in addition to providing background information on interventions prior to and over the drought. Additionally, this study contributes to the considerable gap in the global literature on drought in the context of diarrhoeal disease. This study is also highly relevant as South Africa is predicted to have a hotter and drier climate with severe droughts becoming more likely in the near future [47, 48]. These results are important for the Western Cape as well as other provinces in mitigating the effects of drought, climate change, and climate patterns such as El Niño on health. In terms of study limitations, our data focuses only on the public healthcare system and our interview participants were also mostly governmental officials, many of which were aware of diarrhoeal surge season activities. In both the quantitative and qualitative sections, we were not able to access the private health system, which limits our ability to understand full picture of diarrhoeal disease incidence during times of water scarcity. Additionally, the diarrhoeal disease dataset was aggregated and did not contain any socioeconomic data. It additionally relies on reporting to primary health care facilities, although we tried to mitigate this bias by only looking at cases with dehydration. Finally, the location of the weather stations may have a large impact on regression results disaggregated to the subdistrict level; no weather stations are located in the Cape Flats, a low flat area where many informal settlements are located. Our interviews may not have captured all views, especially since we were not able to access the private sector, while speaking with governmental officials required approval and in some cases the government specified who we were allowed to speak with.

## Conclusions

The incidence of diarrhoeal disease with dehydration has markedly decreased between 2010 and 2019, including over the severe drought years. While increasing temperature is one of the main drivers of diarrhoeal disease with dehydration, precipitation, humidity, and extreme climate events like drought have an important role. The decrease in diarrhoeal disease incidence may be partially explained by extensive and sustained diarrhoeal disease interventions inside and outside of the health system during drought. In the broader scheme, the effect of these measures may help to mitigate negative consequences of climate change and extreme weather events. This research contributes to our understanding of the effect of drought on diarrhoeal disease and may be of interest to other areas of South Africa or other drought sensitive regions. Further studies should also look at diarrhoeal disease rates in other provinces, smaller communities facing drought, or over the winter season. When possible, studies should also take into account sociodemographic information of children with diarrhoeal disease in this context. Special focus should also be paid to other impoverished groups such as backyard dwellers.

### Supplementary Information

**Additional file 1: Figure S1.** Code-tree showing the relationship between individual codes, categories, and central themes developed through the in-depth interviews. **Figure S2.** Distribution of temperature, precipitation, and relative humidity by weather stations in Cape Town from January 2010–December 2019. **Figure S3.** Mean monthly temperature, precipitation, and relative humidity by month by weather stations from January 2010–December 2019. **Figure S4.** Incidence rate of diarrhoeal disease with dehydration in children under five years plotted alongside weather from January 2010–December 2019.

**Additional file 2: Table S1.** Codebook developed for in-depth interview analysis. **Table S2.** Framework analysis matrix for in-depth interviews.

## Data Availability

Interview data generated and analysed during the current study are not publically due to the presence of confidential participants’ information but are available from the corresponding author on reasonable request. The climate and diarrhoeal incidence data that support the findings of this study are available from the SAWS and the Western Cape DoH but restrictions apply to the availability of these data, which were used under license for the current study, and so are not publicly available. Data are however available from the authors upon reasonable request and with permission of both the SAWS and the Western Cape DoH.

## Abbreviations

*CI*: Confidence interval
*IRR*: Incidence rate ratio
*PHC*: Public healthcare
SAWS: South African Weather Service
SINJANI database: Standard Information Jointly Assembled by Networked Infrastructure database
WaSH: Water, Sanitation, and Hygiene
