# Supplementary material for: Understanding diarrhoeal diseases in response to climate variability and drought in Cape Town, South Africa: a mixed methods approach
Source: Infect Dis Poverty. 2023 Aug 18;12:76. doi: 10.1186/s40249-023-01127-7 (PMC10436439; doi:10.1186/s40249-023-01127-7)
Supplement: Supplementary file 1 — Additional file 1: Figure S1. Code-tree showing the relationship between individual codes, categories, and central themes developed through the in-depth interviews. Figure S2. Distribution of temperature, precipitation, and relative humidity by weather stations in Cape Town from January 2010–December 2019. Figure S3. Mean monthly temperature, precipitation, and relative humidity by month by weather stations from January 2010–December 2019. Figure S4. Incidence rate of diarrhoeal disease with dehydration in children under five years plotted alongside weather from January 2010–December 2019. [file 40249_2023_1127_MOESM1_ESM.docx]

Supplementary Information


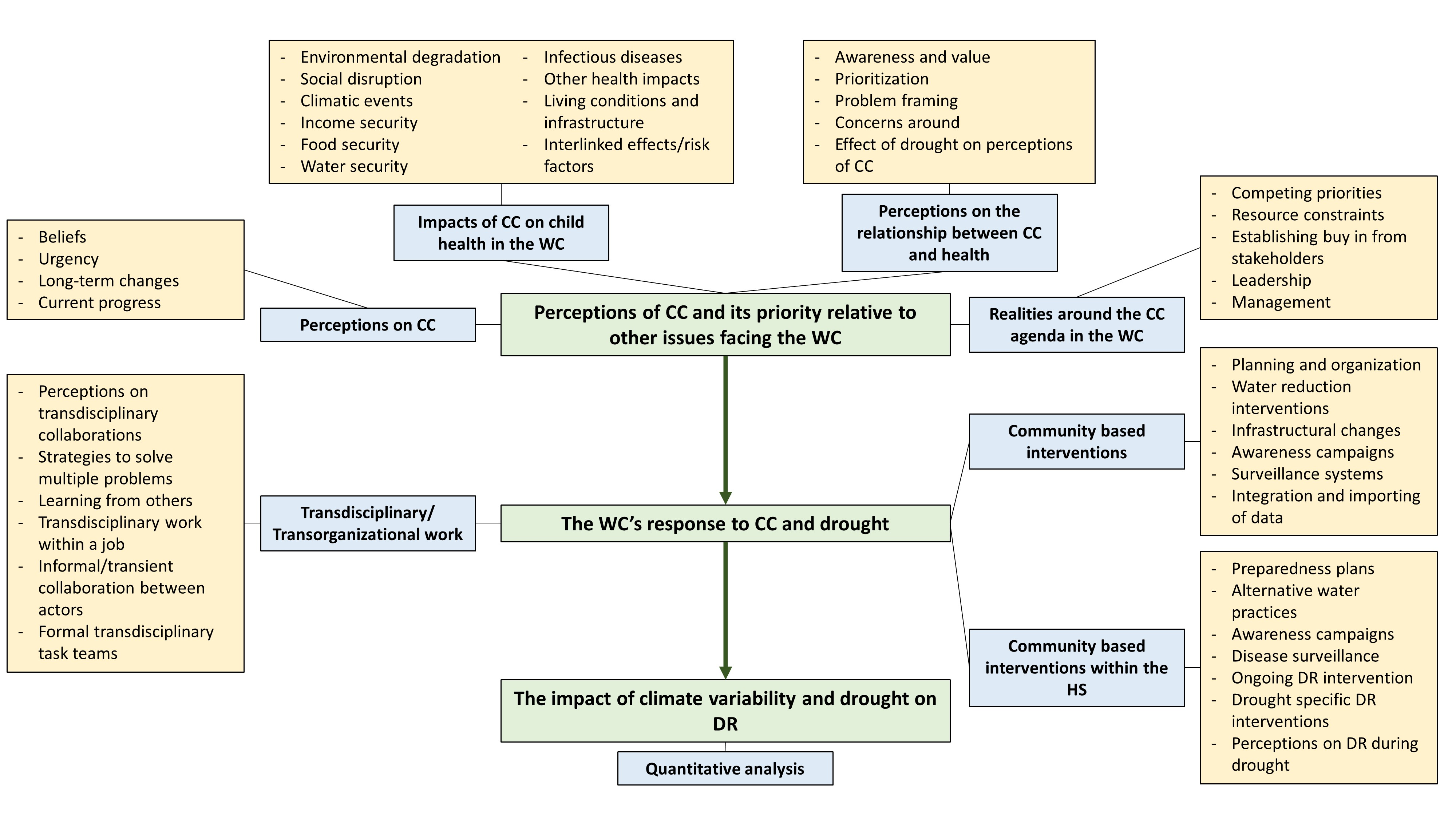


Figure S1. Code-tree showing the relationship between individual codes, categories, and central themes developed through the in-depth interviews. Not all interview data is presented in this manuscript. CC = Climate Change, DR = Diarrhoeal Diseases, HS = Health System, WC = Western Cape


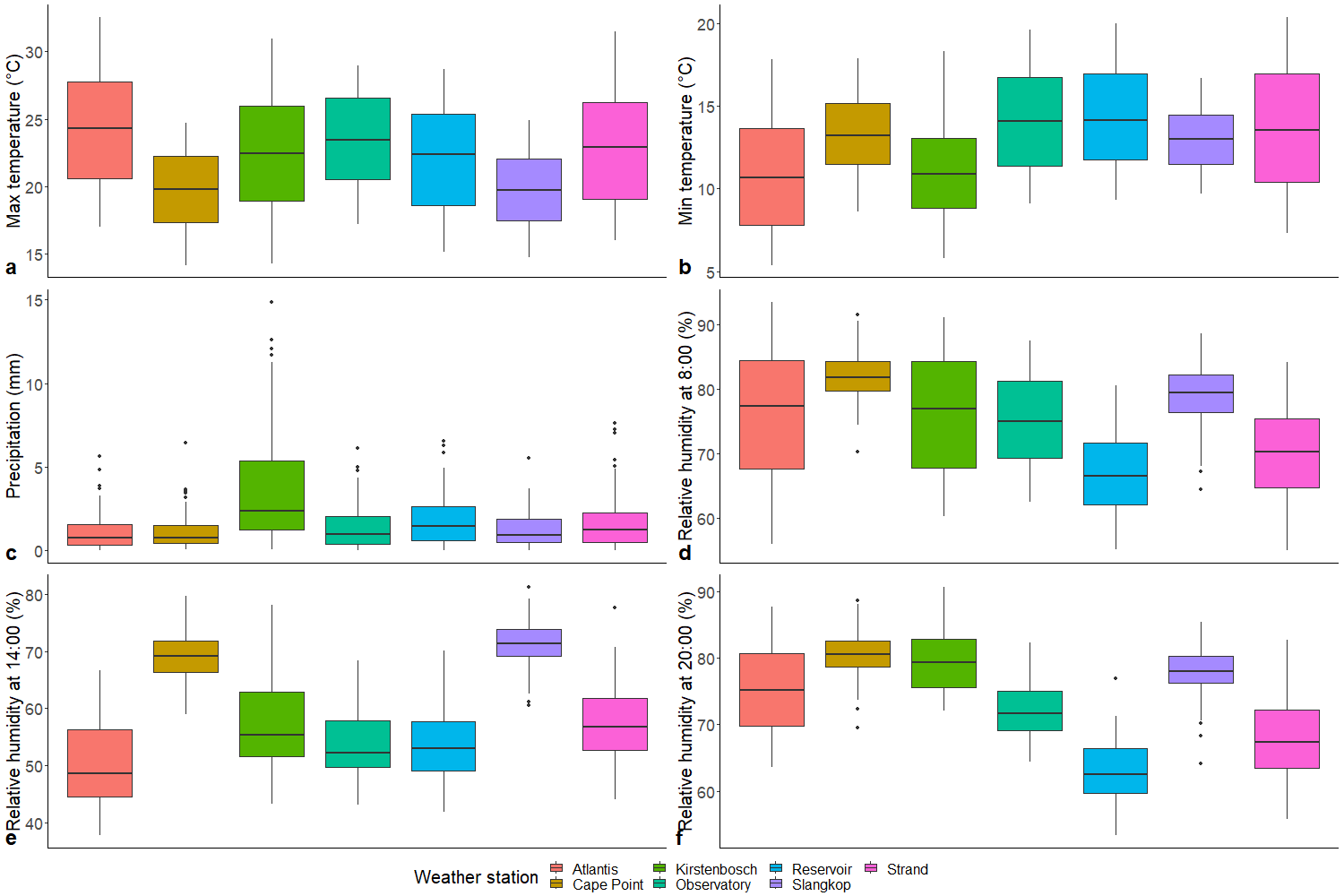


Figure S2. Distribution of temperature, precipitation, and relative humidity by weather stations in Cape Town from January 2010 – December 2019. Values for the stations are summarised by averaging daily measurements from January 2010 – December 2019. a) Mean maximum temperature, b) mean minimum temperature, b) mean precipitation, d) mean relative humidity at 8:00h, e) mean relative humidity at 14:00h, and f) mean relative humidity at 20:00h.


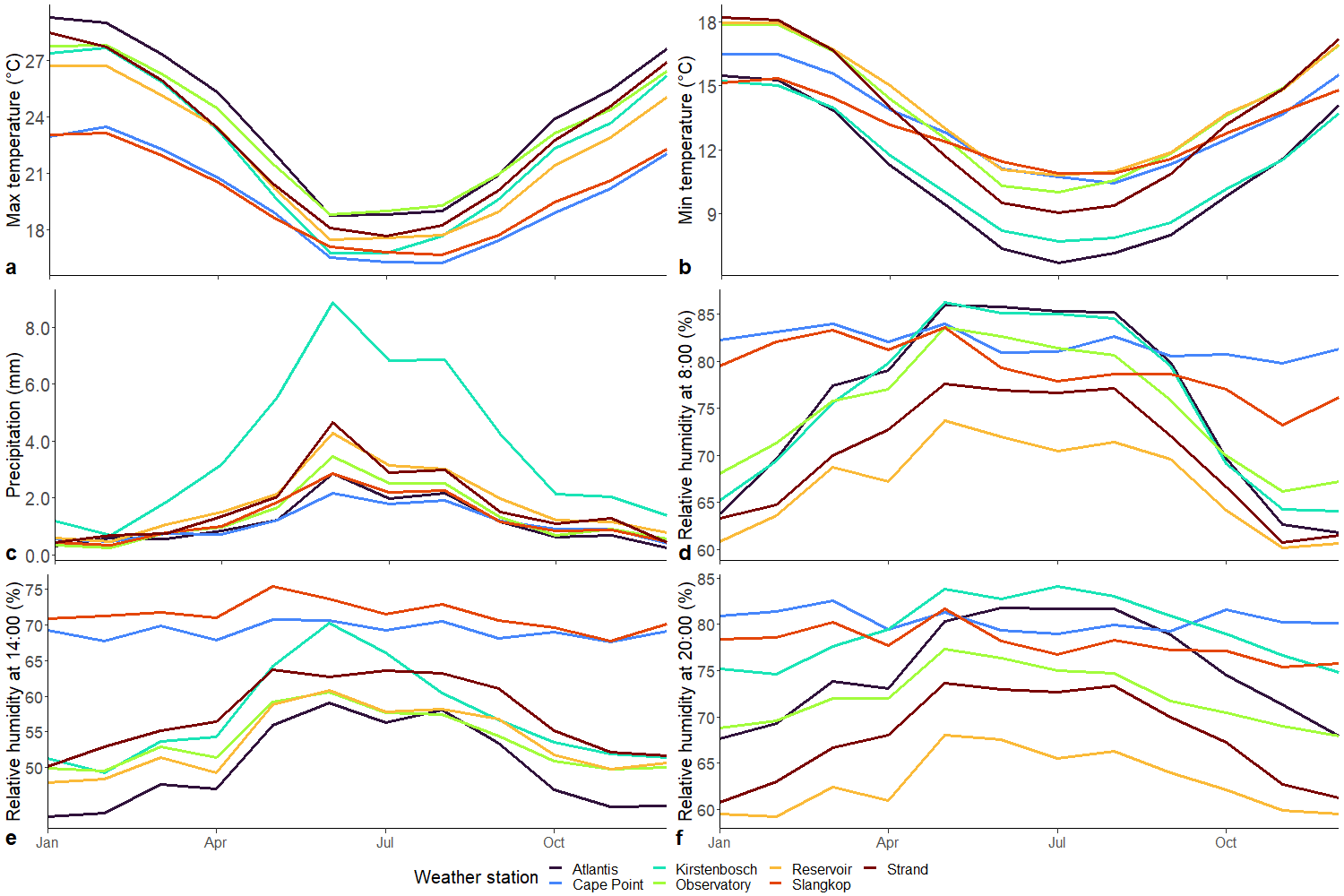


Figure S3. Mean monthly temperature, precipitation, and relative humidity by month by weather stations from January 2010 – December 2019. Monthly values are summarised by averaging daily measurements for a) mean maximum temperature, b) mean minimum temperature, c) mean precipitation, d) mean relative humidity at 8:00h, e) mean relative humidity at 14:00h, and f) mean relative humidity at 20:00h.


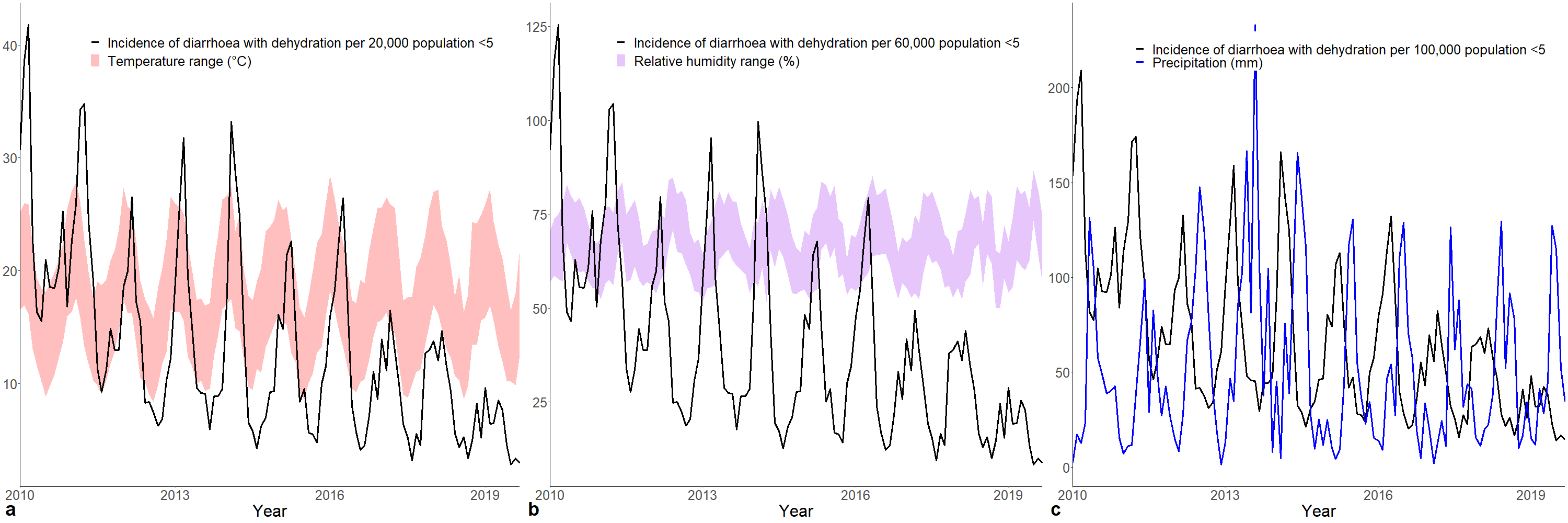


Figure S4. Incidence rate of diarrhoeal disease with dehydration in children under five years plotted alongside weather from January 2010 – December 2019 for a) temperature, b) relative humidity, and c) precipitation. Monthly values are summarised by averaging daily measurements. Temperature and relative humidity ranges are derived from the monthly mean minimum and maximum values of temperature and relative humidity (relative humidity at 14:00 and relative humidity at 8:00), respectively.
